# Baseline Serum Vitamin A and D Levels Determine Benefit of Oral Vitamin A&D Supplements to Humoral Immune Responses Following Pediatric Influenza Vaccination

**DOI:** 10.3390/v11100907

**Published:** 2019-09-30

**Authors:** Nehali Patel, Rhiannon R. Penkert, Bart G. Jones, Robert E. Sealy, Sherri L. Surman, Yilun Sun, Li Tang, Jennifer DeBeauchamp, Ashley Webb, Julie Richardson, Ryan Heine, Ronald H. Dallas, A. Catharine Ross, Richard Webby, Julia L. Hurwitz

**Affiliations:** 1 Department of Infectious Diseases, St. Jude Children’s Research Hospital, Memphis, TN 38105, USA; nehali.patel@stjude.org (N.P.); rhiannon.penkert@stjude.org (R.R.P.); bart.jones@stjude.org (B.G.J.); bob.sealy@stjude.org (R.E.S.); sherri.surman@stjude.org (S.L.S.); jennifer.deBeauchamp@stjude.org (J.D.); ashley.webb@stjude.org (A.W.); ryan.heine@stjude.org (R.H.); ronald.dallas@stjude.org (R.H.D.); richard.webby@stjude.org (R.W.); 2 Department of Biostatistics, St. Jude Children’s Research Hospital, Memphis, TN 38105, USA; yilun.sun@stjude.org (Y.S.); li.tang@stjude.org (L.T.); 3 Department of Pharmaceuticals, St. Jude Children’s Research Hospital, Memphis, TN 38105, USA; julie.richardson@stjude.org; 4 Department of Nutritional Sciences, Pennsylvania State University, University Park, PA 16802, USA; acr6@psu.edu; 5 Department of Microbiology, Immunology and Biochemistry University of Tennessee Health Science Center, Memphis, TN 38163, USA

**Keywords:** influenza virus vaccine, pediatric, vitamins A and D, baseline, antibody response, supplement

## Abstract

Maximizing vaccine efficacy is critical, but previous research has failed to provide a one-size-fits-all solution. Although vitamin A and vitamin D supplementation studies have been designed to improve vaccine efficacy, experimental results have been inconclusive. Information is urgently needed to explain study discrepancies and to provide guidance for the future use of vitamin supplements at the time of vaccination. We conducted a randomized, blinded, placebo-controlled study of influenza virus vaccination and vitamin supplementation among 2 to 8 (inclusive) year old children over three seasons, including 2015–2016 (*n* = 9), 2016–2017 (*n* = 44), and 2017–2018 (*n* = 26). Baseline measurements of vitamins A and D were obtained from all participants. Measurements were of serum retinol, retinol-binding protein (RBP, a surrogate for retinol), and 25-hydroxyvitamin D (25(OH)D). Participants were stratified into two groups based on high and low incoming levels of RBP. Children received two doses of the seasonal influenza virus vaccine on days 0 and 28, either with an oral vitamin supplement (termed A&D; 20,000 IU retinyl palmitate and 2000 IU cholecalciferol) or a matched placebo. Hemagglutination inhibition (HAI) antibody responses were evaluated toward all four components of the influenza virus vaccines on days 0, 28, and 56. Our primary data were from season 2016–2017, as enrollment was highest in this season and all children exhibited homogeneous and negative HAI responses toward the Phuket vaccine at study entry. Responses among children who entered the study with insufficient or deficient levels of RBP and 25(OH)D benefited from the A&D supplement (*p* < 0.001 for the day 28 Phuket response), whereas responses among children with replete levels of RBP and 25(OH)D at baseline were unaffected or weakened (*p* = 0.02 for the day 28 Phuket response). High baseline RBP levels associated with high HAI titers, particularly for children in the placebo group (baseline RBP correlated positively with Phuket HAI titers on day 28, *r* = 0.6, *p* = 0.003). In contrast, high baseline 25(OH)D levels associated with weak HAI titers, particularly for children in the A&D group (baseline 25(OH)D correlated negatively with Phuket HAI titers on day 28, *r* = −0.5, *p* = 0.02). Overall, our study demonstrates that vitamin A&D supplementation can improve immune responses to vaccines when children are vitamin A and D-insufficient at baseline. Results provide guidance for the appropriate use of vitamins A and D in future clinical vaccine studies.

## 1. Introduction

Vitamin A and D metabolites function as nuclear hormones and have profound influences on innate and adaptive immune activities [1,2,3,4,5,6]. These micronutrients have each been shown to correlate positively with immune responses in a portion of small animal and clinical studies [1,2,3,7,8,9,10,11,12,13]. Vitamin A, for example, was described by Rahman et al. to improve immune responses toward a diptheria vaccine [14]. Chadha et al. showed that vitamin D correlated positively with immune responses toward influenza virus in a set of cancer patients [15] and others reported that vitamin D could control a variety of infectious pathogens including influenza viruses in humans [9,16,17,18]. Vitamins A and D function in part by binding nuclear hormone receptors. Receptors include the retinoic acid receptor [RAR], the peroxisome proliferator-activated receptor β/δ [PPAR β/δ], and the vitamin D receptor [VDR], each complexed with retinoid X receptor (RXR) as a heterodimer [19,20]. Receptors regulate gene expression by binding nuclear hormone response elements (NHRE) in mammalian DNA [8,21,22,23,24,25,26,27]. Recently, we discovered NHRE within key promoter, enhancer, and switch sites in immunoglobulin gene loci. We found that nuclear hormone receptors could bind these sites, indicating a direct influence of nuclear hormones on antibody expression [28,29,30,31].

Despite the positive influences described above, clinical studies have yielded disparate results [2,3,4,6,7,8,14,32,33,34,35,36,37,38,39,40,41,42,43,44,45,46,47,48]. When Hanekom et al. tested vitamin A therapy in HIV-infected individuals, they identified no improvements in responses toward influenza virus vaccines [43] and when Lee et al. tested correlations between vitamin D levels and antibody titers after influenza virus vaccinations, significant correlations were not found [49]. An influenza virus vaccine study by Sundaram et al. revealed no consistent association between vitamin D levels and the vaccine-induced antibody response in older adults, although there was a greater frequency of post-vaccination ‘sero-protection’ against H1N1 among vitamin D deficient individuals in the first year of the study [50].

A lack of clarity as to when and how vitamins influence immune responses toward vaccines is concerning because vitamin A and D deficiencies and insufficiencies are prevalent worldwide, affecting both developed and developing countries [11]. Low vitamin levels render individuals vulnerable to infectious diseases [9,13]. Although vitamin supplementation programs are common in developing countries where deficiencies are a known public health concern, they cannot be implemented worldwide without a comprehensive understanding of benefits and risks. 

Surprisingly, although there is considerable interest in the independent effects of vitamins A and D on the immune response, the two vitamins are rarely examined together. This is despite the knowledge that vitamins A and D influence numerous cell functions, are closely related, and can be cross-regulated [8,20,24,26,27,51,52,53,54,55,56,57,58]. Unfortunately, when clinical studies are designed to evaluate the influences of vitamin supplements on the immune response, the baseline vitamin A and/or D levels of study participants are often unknown, and the potential consequences of vitamin cross-regulation are rarely considered. 

To address knowledge gaps, we designed a pediatric randomized controlled study of influenza virus vaccination that measured baseline vitamin A (using retinol-binding protein [RBP] as a surrogate [59]) and 25(OH)D levels among healthy children. Participants were stratified into two groups based on high and low baseline RBP levels and randomized to receive a vitamin A and D supplement (A&D) or a placebo control immediately prior to influenza virus vaccination. The hemagglutination inhibition (HAI) assay, a standard in the field for the assessment of influenza virus vaccines, was used to measure immune responses [60]. We discovered that baseline vitamin levels were critical parameters that determined not only antibody responses toward the influenza virus vaccine but the influence of vitamin supplementation on the immune response.

## 2. Materials and Methods

### 2.1. Clinical Protocol

#### 2.1.1. Enrollment 

A randomized, placebo-controlled clinical study was conducted at St. Jude Children’s Research Hospital (St. Jude) in Memphis, TN to examine the influences of baseline vitamin levels and vitamin supplementation on immune responses to the influenza virus vaccine (https://clinicaltrials.gov/ct2/show/NCT02649192). The study was conducted in accordance with the Declaration of Helsinki, and the protocol (FluVIT, Pro00006109, 5 December 2015) was approved by the Institutional Review Board of St. Jude. Healthy children between 2 to 8 (inclusive) years of age were enrolled over three influenza virus seasons including season 2015–2016 (*n* = 9), season 2016–2017 (*n* = 44), and season 2017–2018 (*n* = 26). Informed consent was given by parents or guardians and assent was given by minors when age-appropriate. Comprehensive histories of previous vaccinations and influenza virus exposures were not available. Participants were excluded from study entry if they had any chronic illness, developmental delay, or neurological disorder. They were also excluded if they were known to have received an influenza vaccine for the current season or were routinely taking a daily vitamin supplement.

#### 2.1.2. Randomization and Masking

During the screening visit, sera were collected for measurements of baseline serum RBP (usually present in blood at a 1:1 molar ratio with retinol [59]) for stratification of participants into “high” and “low” groups. Stratification to the “high” (vitamin A sufficient) group was based on a measurement of ≥ 22,000 ng/mL RBP (approximating ≥ 1.05 µM retinol) [59,61,62,63]. Each group was then randomized to receive A&D as an oral gummy vitamin supplement (20,000 IU and 2000 IU per gummy for vitamins A and D, respectively) or a matched gummy that lacked vitamins (placebo). Stratification was performed to prevent an imbalance between placebo and A&D study groups. Gummies were formulated by Regel PharmaLab (Memphis, TN, USA). The components were vitamin A palmitate liquid (#30-3124, PCCA USA, Houston, TX, USA), vitamin D3 liquid (#30-1033, PCCA USA), gelatin base (#30-1520, 830 mg/dose, PCCA USA), tangerine oil flavor (#30-2155, 1µL/dose, PCCA USA), steviol glycosides 95% (8.7 mg/dose, PCCA USA), citric acid USP anhydrous fine granular (12.5 mg/dose, Letco Medical LLC, Decatur, AL, USA), polysorbate 20 NF liquid (2 µL/dose, PCCA USA), and silica gel micronized powder (4 mg/dose, Letco). Gummies were administered to participants on day 0 and day 28 prior to influenza virus vaccination. Participants and healthcare providers were blinded throughout the vaccination and collection procedures.

#### 2.1.3. Vaccine Components

In season 2015–2016, the vaccine was FluMist^®^ (AstraZeneca, Cambridge, UK), whereas in seasons 2016–2017 and 2017–2018, the vaccines were Fluzone^®^ Quadrivalent (Sanofi Pasteur, Lyon, France) for children < 3 years of age and Fluarix^®^ Quadrivalent (GlaxoSmithKline, Brentford, UK) for children of ≥ 3 years of age. These changes were due to revised recommendations by the Advisory Committee on Immunization Practices (ACIP) of the Centers for Disease Control and Prevention (CDC). Vaccine components changed in each influenza virus season. In 2015–2016, components were A/CA/7/09 H1N1, A/Switzerland/9715293/13 H3N2, B/Phuket/3073/13, and B/Brisbane/60/2008. In 2016–2017 components were A/CA/7/09 H1N1, A/Hong Kong/4801/14 H3N2, B/Phuket/3073/13, and B/Brisbane/60/2008, and in 2017–2018, components were A/Michigan/45/2015 H1N1, A/Hong Kong/4801/14 H3N2, B/Phuket/3073/13, and B/Brisbane/60/2008.

#### 2.1.4. Blood Sample Collection

Blood samples were collected at screening (≤10 days from day 0) and on days 0, 28, and 56. Participants who completed the day 28 visit were considered evaluable.

### 2.2. RBP, Retinol, and 25(OH)D Measurements

Blood samples were preserved in the cold and dark according to standard guidelines. Levels of RBP were measured with an enzyme-linked immunosorbent assay (ELISA, R&D Systems [Minneapolis, MN, USA] human RBP4 Quantikine kits).

Retinol was measured by extraction from test samples under conditions of UV-blocked lighting with samples of SRM-968f human reference sera (NIST, https://www-s.nist.gov/srmors/orderingSRMs.cfm) used as controls. Samples were diluted with HPLC-grade water and then absolute ethanol and HPLC grade hexane. Tubes were vortexed, incubated for 30 min in the dark, and centrifuged. Hexane layers were removed. A second hexane extraction followed, which was combined with the first, and evaporated to dryness under nitrogen. Methanol:water:acetonitrile (10:20:70 by volume) was added followed by vortexing. Samples were transferred to UPLC vials, briefly centrifuged, and loaded into a Waters Acquity UPLC tray for injection onto a Waters Acquity BEH C-18 reverse phase column using methanol:water:acetonitrile (10:20:70) as the mobile phase. Standards of purified all-trans-retinol (0 to 0.8 pmol/10 µL) provided a standard curve (*R*^2^ > 0.99).

25(OH)D was tested in the Pathology Department at St. Jude using the Roche Elecsys Vitamin D ELISA (Roche, Basel, Switzerland) that measures 25(OH)D metabolites of cholecalciferol (vitamin D3) and ergocalciferol (vitamin D2). Vitamin D sufficiency was defined as ≥ 30 ng/mL 25(OH)D.

### 2.3. Hemagglutination Inhibition (HAI) Assay

The HAI assay was conducted to evaluate antibody responses to each of the 4 components in the seasonal influenza virus vaccine. Briefly, antigen (~4 agglutination doses representing each antigen) was added to wells of a 96-well plate containing serial dilutions of test sera (initiated with a 1:10 serum dilution followed by serial 1:2 dilutions, each tested in duplicate). After 30 min incubation at room temperature, 50 microliters of 0.5% *vol*/*vol* turkey red blood cells were added to each well and plates were incubated at room temperature for an additional 30 min. Titers were recorded as the highest dilution that inhibited hemagglutination. A few participants did not return for the day 56 visit. A score of 5 (1/2 the lowest dilution tested) was given if no HAI activity was detected. If two different values were observed for duplicate samples, the geometric mean value was used for graphing and calculation purposes.

### 2.4. Statistical Analyses

Results for each vaccine season were tested independently due to differences in vaccine composition each year. Spearman’s rank correlation was applied to evaluate relationships involving ordinal variables. A generalized estimating equation (GEE) model was used to assess the effect of influenza vaccine plus A&D over time on HAI responses to each antigen among participants with different baseline vitamin levels. Specifically, the GEE model was constructed with log2-transformed HAI titers as the response, age, race, and a three-way interaction among time, baseline vitamin levels, and study groups as covariates, and the first order autoregressive (AR1) as the working correlation structure. Data analyses were performed using SAS 9.4 (SAS Institute, Inc, Cary, NC, USA) and GraphPad Prism software (San Diego, CA, USA).

## 3. Results

### 3.1. Participant Characteristics

Age, sex, race, and baseline vitamin levels are shown for all three seasons and all participants in Table 1. As shown, a different vaccine was administered to study participants each year, and FluMist^®^ was only used in season 2015–2016 due to revised recommendations by the CDC. As expected, baseline RBP and retinol levels were positively correlated (Spearman’s correlation *r* = 0.7, *p* < 0.0001), emphasizing that RBP can serve as a surrogate for retinol. An additional test of RBP was conducted on day 56 for placebo and A&D groups, but day 56 values were not significantly different from day 0 values in either group. Baseline HAI means and ranges are shown for each season (Table 1). For HAI, data were not combined among years because vaccines and consequent immune responses differed. Full details for all participants and all years are provided in Table A1, Table A2, Table A3, Table A4, Table A5 and Table A6.

Table 2 shows the frequencies of ≥ 4-fold increases in HAI titers by year and influenza virus antigen on days 28 and 56. As shown, the largest participant number was in season 2016–2017 (*n* = 44). Accordingly, results from this season were used for the primary analyses. For other seasons, descriptive analyses were performed due to limited available data.

For several vaccine components, baseline HAI titers were highly variable among study participants, a situation that may confound comparisons between participants and study groups. Fortuitously, for the B/Phuket/3073/13 vaccine in the 2016–2017 season, when enrollment was highest, baseline titers were all negative (highlighted in Table 1).

### 3.2. Vitamin Supplements were Beneficial to Children with Insufficient Vitamin A and D Levels at Baseline

**Figure 1:**
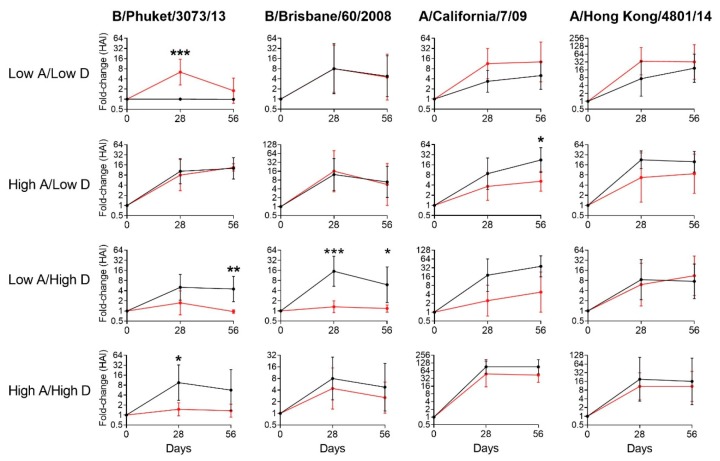
Estimated fold change in hemagglutination inhibition (HAI) responses compared between placebo (black) and vitamin A and D supplement (A&D, red) study groups at different time points of the study in season 2016–2017. Participants were placed into one of four groups based on vitamin levels. Cut-offs for sufficiency (termed “high”) were ≥ 22,000 ng/mL for retinol-binding protein (RBP) and ≥ 30 ng/mL for 25(OH)D. Groups were “Low A/Low D”, *n* = 10; “High A/Low D”, *n* = 12; “Low A/High D,” *n* = 11; “High A/High D,” *n* = 11 (see Table 2). For each of the four viruses, the GEE model was constructed with log2-transformed HAI titers as the response, age, race, and a three-way interaction among time, incoming vitamin levels, and study groups as covariates, and the first order autoregressive (AR1) as the working correlation structure. Estimated mean fold changes are plotted with 90% confidence intervals. *p*-values were obtained from post hoc comparisons from GEE models (* *p* < 0.05, ** *p* < 0.01, *** *p* < 0.001).

To determine how baseline vitamin levels affected immune responses and benefits of vitamin supplementation, we characterized each participant from season 2016–2017 as being sufficient or insufficient/deficient for vitamins A or D at baseline, and accordingly assigned participants to one of four groups (e.g., the “Low A/Low D” group included individuals who were insufficient or deficient for both vitamins A and D). Cut-offs for sufficiency (termed “high”) were ≥ 22,000 ng/mL for RBP and ≥ 30 ng/mL for 25(OH)D. The fold-change of HAI titers on days 28 and 56 compared to baseline were examined for each group (Table 2 and Figure 1.) The percentages of participants with a ≥ 4-fold rise in HAI activity on day 28 or day 56 compared to baseline are shown in Table 2. Of particular interest, we found that children who were insufficient or deficient for both vitamins A and D (Low A/Low D) benefitted significantly from the A&D supplement (Table 2 and Figure 1.).

### 3.3. Vitamin Supplements were Ineffective or Inhibitory of Vaccine Responses among Children with Sufficient Levels of Vitamins A and/or D

The benefit of A&D supplementation differed for children with sufficient levels of vitamins A and/or D at baseline compared to children who were insufficient or deficient. As shown in Figure 1, A&D benefits were not observed when children exhibited sufficient vitamin levels at baseline. In fact, for some responses, the A&D supplement conferred an inhibitory effect on HAI responses. Results help explain discrepancies in previous clinical studies by showing that the benefit of a vitamin supplement can be determined by the study participant’s baseline vitamin levels.

### 3.4. Baseline RBP Correlates Positively, while Baseline 25(OH)D Correlates Negatively, with Immune Responses Toward Influenza Vaccine Components.

**Figure 2:**
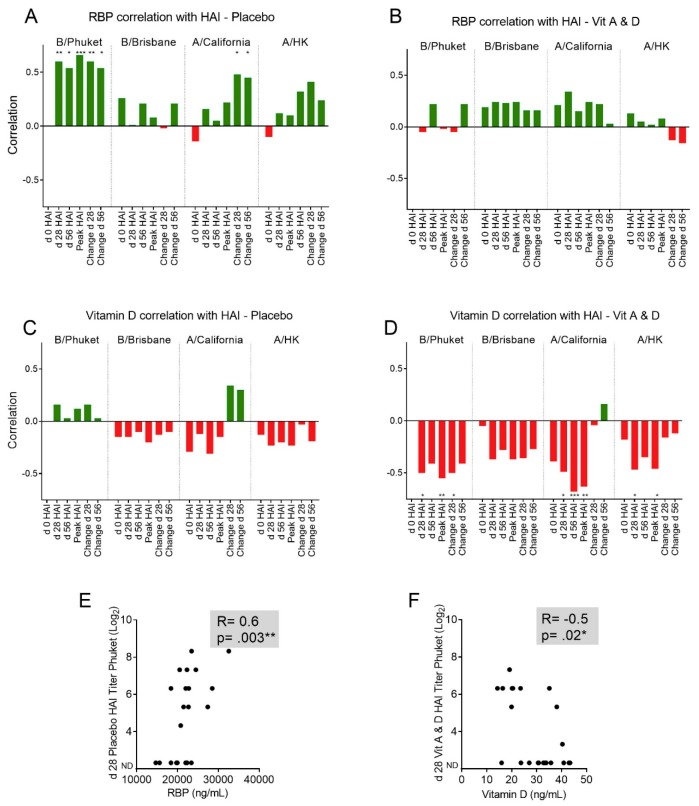
Relationships between baseline vitamin levels and HAI responses in season 2016–2017. Spearman correlation coefficients are plotted for comparisons between baseline vitamin levels and HAI titers among participants enrolled during the 2016–2017 season. R values are plotted on the Y axis in A–D. Positive correlations are indicated in green and negative correlations are indicated in red. The top row shows RBP correlations with HAI titers in placebo (**A**) and A&D (**B**) groups. Specifically, correlations are shown between baseline RBP and day 0 HAI, baseline RBP and day 28 HAI, baseline RBP and day 56 HAI, baseline RBP and peak HAI, baseline RBP and changes in HAI between days 0 and 28, and baseline RBP and changes in HAI between days 0 and 56. * *p* < 0.05; ** *p* < 0.01; *** *p* < 0.001. The middle row substitutes 25(OH)D for RBP in placebo (**C**) and A&D (**D**) groups. In the bottom row, detailed correlative data are shown for RBP versus B/Phuket/3073/13 HAI titers on day 28 in the placebo group (*n* = 22, some values overlap) (**E**), and 25(OH)D versus B/Phuket/3073/13 HAI titers on day 28 in the A&D group (*n* = 22, some values overlap) (**F**).

We next examined how baseline serum vitamin A and D levels correlated with HAI toward the four vaccine components in season 2016–2017 (A/CA/7/09 H1N1, A/Hong Kong/4801/14 H3N2, B/Phuket/3073/13, and B/Brisbane/60/2008). Correlations were evaluated between baseline RBP levels and HAI titers (including absolute HAI titers on day 28 and day 56, peak HAI titers, or changes in HAI titers between days 0 and 28 or days 0 and 56). As shown in Figure 2A and 2B, baseline RBP was positively correlated with HAI.

This result was more pronounced in the placebo group (Figure 2A) compared to the A&D group. Surprisingly, the same result was not observed for 25(OH)D. In this case, there were often negative correlations between 25(OH)D and the HAI immune response (i.e., a high 25(OH)D level at study entry associated with a low HAI response).

The negative correlation was more pronounced in the A&D group (Figure 2D) compared to the placebo group. Individual patient data for two of these correlations are shown in Figure 2E–F. As shown, the baseline RBP value was positively and significantly correlated with the day 28 HAI response toward B/Phuket/3073/13 in the placebo group (*r* = 0.6, *p* = 0.003, Figure 2E), and the baseline 25(OH)D value was negatively and significantly correlated with the day 28 HAI response toward B/Phuket/3073/13 in the A&D group (*r* = −0.5, *p* = 0.02, Figure 2F).

## 4. Discussion

Our clinical study was designed to investigate how baseline vitamins A and D influence the immune response to influenza virus vaccines. We found that baseline vitamin A levels (scored by RBP) correlated positively with immune responses, particularly toward the B/Phuket/3073/13 vaccine component in the placebo group in season 2016–2017. However, negative correlations were observed between baseline 25(OH)D and the immune response, particularly in the group that received vitamin supplementation. The vitamin A&D supplement significantly improved immune responses toward the B/Phuket/3073/13 vaccine in season 2016–2017 but only when baseline vitamin A and D levels were insufficient at baseline. The supplement had no significant effect or weakened the HAI response when baseline RBP and 25(OH)D levels were sufficient.

Our results were somewhat surprising given that vitamins are often viewed as positively associated with immune responses. Our observation that 25(OH)D correlated negatively with vaccine-induced HAI appeared contrary to the report by Chadha et al. who observed a positive influence of vitamin D on the immune response to influenza virus in cancer patients [15]. Of note, not all researchers have agreed that vitamins are beneficial. Some authors have argued that “too much” vitamin can have a negative influence on the immune response to vaccines [5,34,40,44,45]. Lee et al. (described above), Principi et al. [41], and Kriesel et al. [64] each failed to identify an influence of vitamin D on influenza virus-specific responses. In the latter two studies vitamin D supplements were administered to influenza virus vaccine participants but supplements did not improve vaccine-induced immune responses. A study by Lin et al. [45] showed a negative association between vitamin D levels and responses to type B influenza viruses. Our data help to explain the differing results and interpretations of previous literature by showing that baseline vitamin A and baseline vitamin D levels each affect outcome; we found that the benefit of a vitamin A&D supplement was only evident when children were insufficient or deficient in both RBP and 25(OH)D at study entry.

To explain why 25(OH)D could have a negative influence on immune responses to influenza virus vaccines, we consider the vitamin’s capacity to clear pathogens. Vitamin D upregulates cathelicidin, an anti-microbial peptide [65,66] that can denature and clear both bacteria and viruses, including influenza virus. Perhaps children who were 25(OH)D replete (due to diet and/or the A&D supplement) rapidly cleared vaccine antigens, thus removing the trigger for an antibody response. The influenza virus vaccine is not adjuvanted and may have been particularly vulnerable to cathelicidin-induced damage. A suggestion of rapid vaccine clearance in the presence of high vitamin levels was similarly proposed by Semba et al. when their vitamin A supplementation of 6 month old infants was found to inhibit antibody responses to the measles vaccine [44]. Another explanation for vitamin D inhibition of HAI may relate to the vitamin’s capacity to alter innate and adaptive immune cell trafficking in vivo, and to inhibit B cell proliferation and survival in vitro [1,67]. One final consideration concerns the contrasting influences of vitamins A and D; vitamin D has been positively correlated with serum IgM and IgG3, whereas vitamin A has been positively correlated with other isotypes that might better support HAI [11,68,69,70]. If vitamin D and its receptor blocked the binding of RAR to DNA response elements, vitamin D may have inhibited positive influences of vitamin A [27].

Our study had limitations because more than one factor could have influenced HAI responses and data interpretation. The phenotypes and functions of individual T cell populations, B cell populations and innate immune cells were not analyzed in our study. T cell and antigen presenting cell (APC) populations are particularly important as drivers of B cell activation, proliferation, and antibody expression. Each can be influenced by vitamin levels. In vitamin A deficient mice, for example, CD103 is significantly upregulated on dendritic cell (DC) and T cell membranes [1,12]. CD103 is the αE component of the αEβ7 integrin, which binds the epithelial cell marker E-cadherin. Changes in homing receptor expression affect DC and T cell trafficking/residence and thereby alter antigen presentation and the “help” T cells provide to virus-specific B cells [1,12,71]. The plethora of indirect and direct influences of nuclear hormones on the B cell response (including nuclear hormone receptor binding to enhancers and switch regions of the immunoglobulin heavy chain locus [28,29,58]) may explain some of the complex outcomes of clinical studies. Our primary data were from season 2016–2017 with the highest enrollment; smaller datasets from other seasons were sufficient only for observation and not for confirmatory analyses. We note differences between HAI responses to the four components in each vaccine. For example, among individuals with low baseline RBP and low baseline 25(OH)D levels in the 2016–2017 season, the most significant improvements in vaccine-induced responses in the A&D group compared to the placebo group were toward the B/Phuket/3073/13 vaccine component. In this case, our analyses of vaccine-induced immune responses benefitted from the harmonious and negative HAI responses toward B/Phuket/3073/13 among study participants at baseline; in contrast, study participants exhibited variable and confounding baseline responses toward the other three vaccine components (Table 1). Another limitation of our study was that subclinical or clinical exposures to cross-reactive viral antigens by study participants before or during vaccination may have contributed to outcomes [72,73]. One individual in the placebo group of the 2016–2017 season (ID #58, Table A2) had a confirmed influenza virus infection and one individual in the placebo group of season 2017–2018 (ID #66, Table A3) reported an exposure to family members with confirmed influenza virus infections.

Despite the limitations noted above, our data provide an explanation for conflicting results in previous vitamin supplementation studies. As in our study, responses to vaccine antigens and vitamin supplements in other studies were likely dependent on baseline vitamin levels [40]. Unfortunately, in past studies, baseline levels of vitamin A, vitamin D, or both, were often untested and/or unreported. Our study provides guidance for the suitability of adding vitamin A and D measurements to future clinical vaccine protocols to improve data interpretation and thereby improve vaccine efficacy and infection control in children. Finally, our results support a recommendation to use vitamin A and D supplements with influenza vaccines in areas where children are frequently vitamin deficient or insufficient but not in areas where children are frequently replete for vitamins A and D.

## Figures and Tables

**Table 1:** Baseline patient characteristics.

Characteristics	All Seasons FluMist, Fluzone, or Fluarix		2015–2016 Season FluMist		2016–2017 Season Fluzone or Fluarix		2017–2018 Season Fluzone or Fluarix	
	Placebo (*n* = 40)	Vitamin A&D (*n* = 39)	Placebo (*n* = 4)	Vitamin A&D (*n* = 5)	Placebo (*n* = 22)	Vitamin A&D (*n* = 22)	Placebo (*n* = 14)	Vitamin A&D (*n* = 12)
Age groups, *n* (%)								
2–4 years	22 (55 %)	16 (41%)	3 (75%)	1 (20%)	12 (54.5%)	8 (36%)	7 (50%)	7 (58%)
5–8 years	18 (45 %)	23 (59%)	1 (25%)	4 (80%)	10 (45.5%)	14 (64%)	7 (50%)	5 (42%)
Sex, *n* (%)								
Female	23 (57.5%)	23 (59%)	3 (75%)	3 (60%)	14 (64%)	14 (64%)	6 (43%)	6 (50%)
Male	17 (42.5%)	16 (41%)	1 (25%)	2 (40%)	8 (36%)	8 (36%)	8 (57%)	6 (50%)
Race, *n* (%)								
White	11 (27.5%)	11 (28%)	0 (0%)	0 (0%)	6 (27%)	7 (32%)	5 (36%)	4 (33%)
Black	29 (72.5%)	28 (72%)	4 (100%)	5 (100%)	16 (73%)	15 (68%)	9 (64%)	8 (67%)
RBP, *n* (%)								
<22,000 ng/mL	18 (45%)	19 (49%)	2 (50%)	3 (60%)	10 (45.5%)	11 (50%)	6 (43%)	5 (42%)
≥22,000 ng/mL	22 (55%)	20 (51%)	2 (50%)	2 (40%)	12 (54.5%)	11 (50%)	8 (57%)	7 (58%)
Vitamin D, *n* (%)								
<30 ng/mL	21 (52.5%)	21 (54%)	3 (75%)	5 (100%)	12 (54.5%)	10 (45.5%)	6 (43%)	6 (50%)
≥30 ng/mL	19 (47.5%)	18 (46%)	1 (25%)	0 (0%)	10 (45.5%)	12 (54.5%)	8 (57%)	6 (50%)
Retinol, *n* (%)								
<20 µg/dL	2 (5%)	3 (7.7%)	1 (25%)	0 (0%)	1 (4.6%)	2 (9%)	0 (0%)	1 (8.3%)
20–30 µg/dL	11 (27.5%)	8 (20.5%)	3 (75%)	1 (20%)	3 (13.6%)	3 (14%)	5 (36%)	4 (33.3%)
>30 µg/dL	27 (67.5%)	28 (71.8%)	0 (0%)	4 (80%)	18 (81.8%)	17 (77%)	9 (64%)	7 (58.3%)
Baseline HAI titer (log2), median (min, max)								
B/Phuket	---^#^	---	2.32 (2.32, 2.32)	2.32 (2.32, 5.32)	2.32 (2.32, 2.32)	2.32 (2.32, 2.32)	2.32 (2.32, 7.32)	2.32 (2.32, 3.32)
B/Brisbane	---	---	2.32 (2.32, 2.32)	2.32 (2.32, 2.32)	2.32 (2.32, 6.32)	2.32 (2.32, 7.32)	2.32 (2.32, 7.32)	2.32 (2.32, 7.32)
H1N1 ^*^	---	---	3.32 (2.32, 5.32)	2.32 (2.32, 7.32)	3.32 (2.32, 9.32)	2.32 (2.32, 9.32)	7.32 (2.32, 10.32)	7.82 (2.32, 12.32)
H3N2 ^*^	---	---	4.82 (2.32, 8.82)	2.32 (2.32, 9.32)	6.82 (2.32, 10.32)	7.32 (2.32, 11.32)	8.32 (2.32, 10.32)	6.82 (2.32, 10.32)

The numbers and characteristics of study participants in all seasons and in each of the three seasons are shown, with levels for RBP, vitamin D (25(OH)D), and retinol. HAI titers are shown for each season. ^#^HAI titers were not combined among seasons because the vaccine changed each year. In 2015–2016, vaccine components were A/CA/7/09 H1N1, A/Switzerland/9715293/13 H3N2, B/Phuket/3073/13, and B/Brisbane/60/2008. In 2016–2017 components were A/CA/7/09 H1N1, A/Hong Kong/4801/14 H3N2, B/Phuket/3073/13, and B/Brisbane/60/2008, and in 2017–2018 components were A/Michigan/45/2015 H1N1, A/Hong Kong/4801/14 H3N2, B/Phuket/3073/13, and B/Brisbane/60/2008. When an HAI value was below detection, the sample was given a value of 5 (log2 2.32). Tests were conducted in duplicate and geometric means were determined. The negative baseline responses toward B/Phuket/3073/13 among all participants in season 2016–2017 are highlighted. For conversion, 30 µg/dL retinol = 1.05 µmol/L; 20 ng/mL 25(OH)D = 50 nmol/L. ^*^ H1N1 and H3N2 components differed between seasons.

**Table 2:** HAI response (≥4 fold change) after the first or second vaccine doses.

						
	B/Phuket/3073/13		B/Brisbane/60/2008		H1N1 ^*^		H3N2 ^*^	
	Placebo	A&D	Placebo	A&D	Placebo	A&D	Placebo	A&D
**HAI Response** **(≥4 Fold Change) after the 1st Dose (Day 28 HAI Titer Compared to Day 0)**								
2015–2016 (*n* = 9)	0/4 (0%)	1/5 (20%)	0/4 (0%)	0/5 (0%)	0/4 (0%)	0/5 (0%)	2/4 (50%)	2/5 (40%)
2016–2017 (*n* = 44)	13/22 (59%)	9/22 (41%)	17/22 (77%)	10/22 (45%)	16/22 (73%)	14/22 (64%)	16/22 (73%)	15/22 (68%)
2017–2018 (*n* = 26)	1/14 (7%)	3/12 (25%)	3/14 (21%)	4/12 (33%)	10/14 (71%)	8/12 (67%)	9/14 (64%)	10/12 (83%)
2016–2017 (*n* = 44)								
Low A and Low D (*n* = 10)	0/4 (0%)	4/6 (67%)	2/4 (50%)	3/6 (50%)	2/4 (50%)	5/6 (83%)	2/4 (50%)	5/6 (83%)
High A and Low D (*n* = 12)	6/8 (75%)	3/4 (75%)	7/8 (88%)	3/4 (75%)	5/8 (63%)	2/4 (50%)	8/8 (100%)	3/4 (75%)
Low A and High D (*n* = 11)	4/6 (67%)	1/5 (20%)	5/6 (83%)	1/5 (20%)	5/6 (83%)	1/5 (20%)	3/6 (50%)	2/5 (40%)
High A and High D (*n* = 11)	3/4 (75%)	1/7 (14%)	3/4 (75%)	3/7 (43%)	4/4 (100%)	6/7 (86%)	3/4 (75%)	5/7 (71%)
**HAI Response (≥4 Fold Change) after the 2nd Dose (Day 56 HAI Titer Compared to Day 0)**								
2015–2016 (*n* = 9)	0/4 (0%)	0/5 (0%)	0/4 (0%)	0/5 (0%)	0/4 (0%)	0/5 (0%)	2/4 (50%)	2/5 (40%)
2016–2017 (*n* = 41)	11/20 (55%)	6/21 (29%)	12/20 (60%)	6/21 (29%)	19/20 (95%)	15/21 (71%)	16/20 (80%)	15/21 (71%)
2017–2018 (*n* = 25)	4/14 (29%)	5/11 (45%)	5/14 (36%)	3/11 (27%)	12/14 (86%)	8/11 (73%)	7/14 (50%)	8/11 (73%)
2016–2017 (*n* = 41)								
Low A and Low D (*n* = 9)	0/3 (0%)	1/6 (17%)	1/3 (33%)	2/6 (33%)	2/3 (67%)	4/6 (67%)	2/3 (67%)	5/6 (83%)
High A and Low D (*n* = 11)	6/7 (86%)	4/4 (100%)	6/7 (86%)	2/4 (50%)	7/7 (100%)	2/4 (50%)	7/7 (100%)	3/4 (75%)
Low A and High D (*n* = 10)	3/6 (50%)	0/4 (0%)	3/6 (50%)	0/4 (0%)	6/6 (100%)	2/4 (50%)	4/6 (67%)	2/4 (50%)
High A and High D (*n* = 11)	2/4 (50%)	1/7 (14%)	2/4 (50%)	2/7 (29%)	4/4 (100%)	7/7 (100%)	3/4 (75%)	5/7 (71%)

The vitamin group with the greater frequency of responses toward each vaccine component in season 2016–2017 is highlighted. ^*^H1N1 and H3N2 changed between years. A few participants did not return for the day 56 visit.

## Appendix A

**Table A1:** Patient Characteristics 2015–2016.

ID	Sex	Age	Race	Date	RBP (ng/mL)	Retinol (µg/dL)	Vitamin D (ng/mL)	Assn.
1	Female	3	Black	2/11/2016	27,123	26.58	41.66	A
2	Male	7	Black	2/15/2016	25,607	31.36	21.14	B
3	Female	2	Black	2/15/2016	25,056	23.06	23.99	A
4	Female	8	Black	2/16/2016	20,523	30.98	14.39	B
5	Male	4	Black	2/24/2016	12,970	12.15	17.76	A
6	Female	6	Black	2/24/2016	16,248	22.10	24.3	B
7	Female	8	Black	2/24/2016	24,314	37.77	17.16	B
8	Male	4	Black	2/29/2016	21,660	33.59	21.52	B
9	Female	5	Black	2/29/2016	19,773	21.49	22.29	A

Assignments (Assn.) were A = Placebo, B = Vit A&D.

**Table A2:** Patient Characteristics 2016–2017.

ID	Sex	Age	Race	Date	RBP (ng/mL)	Retinol (µg/dL)	Vitamin D (ng/mL)	Assn.
13	Female	8	White	9/26/2016	26,208	31.48	40.32	B
14	Male	7	Black	10/6/2016	19,967	40.69	25.67	A
15	Male	2	Black	10/6/2016	18,628	25.42	40.83	B
16	Male	5	Black	10/14/2016	29,270	40.74	20.46	B
17	Male	7	Black	10/14/2016	22,039	42.72	17.83	A
19	Male	3	Black	10/18/2016	14,717	17.48	17.52	A
20	Female	5	Black	10/21/2016	28,408	33.55	19.94	B
21	Female	2	Black	10/21/2016	22,691	33.41	24.42	A
22	Male	2	White	10/31/2016	22,773	32.63	32.1	A
23	Male	4	Black	11/1/2016	25,284	38.87	38.07	B
24	Female	4	Black	11/1/2016	21,511	34.75	32.94	A
25	Female	8	Black	11/4/2016	28,509	41.73	26.36	A
26	Female	8	Black	11/4/2016	27,407	37.28	31.06	A
27	Male	3	Black	11/2/2016	21,077	34.72	43.38	B
28	Female	8	White	11/10/2016	31,602	45.56	33.12	B
29	Female	5	White	11/10/2016	28,462	36.71	35.72	B
30	Female	8	White	11/18/2016	16,262	37.40	35.09	B
31	Female	6	White	11/18/2016	18,033	34.69	33.81	B
32	Female	7	White	11/18/2016	16,513	27.47	30.6	B
33	Male	2	White	11/18/2016	20,789	32.81	33.9	A
34	Female	3	White	11/22/2016	19,763	30.25	47.91	A
35	Male	7	Black	11/29/2016	21,604	37.01	23.56	B
36	Female	4	Black	12/5/2016	22,322	33.55	18.34	A
38	Female	2	Black	12/13/2016	9,563	18.06	14.29	B
39	Male	3	Black	12/13/2016	18,423	37.10	38.66	A
40	Female	5	Black	12/13/2016	20,554	43.13	41.98	A
41	Female	2	Black	12/29/2016	15,620	29.52	18.44	A
42	Female	8	Black	12/29/2016	23,453	31.06	21.21	A
45	Female	3	Black	1/5/2017	28,088	54.87	27.05	B
46	Female	4	Black	1/5/2017	18,416	30.00	23.72	A
47	Female	3	Black	1/13/2017	23,044	34.92	30.79	B
48	Female	8	Black	1/19/2017	27,113	39.94	19.17	B
49	Female	2	Black	1/20/2017	18,946	26.15	20.18	B
50	Male	6	Black	1/20/2017	21,196	34.16	16.4	B
51	Male	4	Black	1/24/2017	23,402	36.74	20.44	A
53	Female	5	Black	1/26/2017	24,518	35.92	21.74	A
54	Female	6	Black	1/26/2017	22,327	32.28	27.14	A
55	Female	6	Black	1/27/2017	14,551	19.57	23.7	B
57	Male	7	White	2/9/2017	32,563	55.81	31.88	A
58	Female	8	White	2/20/2017	19,934	29.56	31.52	A
60	Female	6	Black	3/3/2017	21,408	33.01	15.95	B
61	Female	4	White	3/7/2017	22,007	31.90	35.13	A
62	Male	5	White	3/7/2017	24,650	30.95	32.71	B
64	Male	2	Black	3/31/2017	28,602	36.90	42.95	B

Assignments (Assn.) were A = Placebo, B = Vit A&D.

**Table A3:** Patient Characteristics 2017–2018.

ID	Sex	Age	Race	Date	RBP (ng/mL)	Retinol (µg/dL)	Vitamin D (ng/mL)	Assn.
66	Male	3	White	9/18/2017	26,710	38.11	33.6	B
67	Female	8	Black	9/20/2017	30,555	43.65	31.28	B
68	Female	8	White	10/13/2017	25,711	37.70	43.25	A
70	Male	2	White	10/20/2017	22,600	34.30	39.52	B
71	Male	8	White	10/20/2017	21,556	43.41	30.74	A
72	Male	2	White	10/20/2017	30,109	36.38	34.36	A
73	Male	2	White	10/20/2017	29,917	41.63	53.05	A
74	Female	2	White	10/30/2017	17,973	26.53	34.74	B
75	Female	2	Black	11/2/2017	22,499	33.34	17.04	B
78	Female	2	Black	11/15/2017	20,178	28.53	56.76	A
79	Male	6	Black	11/16/2017	20,113	34.33	26.05	A
80	Female	8	Black	11/17/2017	16,137	25.51	13.68	B
81	Female	2	Black	11/17/2017	20,112	29.38	23.76	A
83	Male	2	Black	12/5/2017	35,501	47.44	31.49	B
84	Female	8	Black	12/8/2017	15,692	21.53	23.13	A
85	Male	3	Black	12/12/2017	18,809	26.27	28.4	B
86	Male	8	Black	12/12/2017	31,920	44.38	18.75	A
87	Male	8	Black	1/9/2018	37,539	51.88	35.06	B
88	Male	7	Black	1/9/2018	23,074	30.49	37.53	A
89	Female	7	White	2/19/2018	19,055	27.30	25.16	B
90	Female	7	Black	2/20/2018	26,643	38.93	26.8	A
92	Female	8	Black	3/13/2018	30,636	36.42	10.06	B
94	Male	2	Black	3/28/2018	13,348	18.49	20.32	B
95	Female	4	Black	3/26/2018	26,157	29.94	12.97	A
96	Male	2	Black	4/5/2018	20,866	28.02	53.52	A
97	Male	2	White	4/23/2018	25,638	36.52	39.14	A

Assignments (Assn.) were A = Placebo, B = Vit A&D.

**Table A4:** HAI results 2015–2016.

A.	Day 0				Day 28				Day 56			
ID	A/CA/7/09 H1N1	A/Switz/9715293/13 H3N2	B/Phuket/3073/13	B/Brisbane/60/2008	A/CA/7/09 H1N1	A/Switz/9715293/13 H3N2	B/Phuket/3073/13	B/Brisbane/60/2008	A/CA/7/09 H1N1	A/Switz/9715293/13 H3N2	B/Phuket/3073/13	B/Brisbane/60/2008
1	<10	<10	<10	<10	<10	320/640	<10	<10	<10	160	<10	<10
2	<10	640	40	<10	<10	320	160	<10	<10	320	80	<10
3	<10	<10	<10	<10	<10	160	<10	<10	<10	1280	<10	<10
4	160	<10	<10	<10	160	<10	<10	<10	320	<10	<10	<10
5	20	160	<10	<10	<10	320	<10	<10	<10	320	<10	<10
6	<10	<10	<10	<10	<10	1280	<10	<10	<10	1280	<10	<10
7	40	160/80	<10	<10	<10	<10	<10	<10	<10	80	<10	<10
8	<10	<10	<10	<10	<10	640	<10	<10	<10	320	<10	<10
9	40	320/640	<10	<10	40	320	<10	<10	<10	640	<10	<10

**Table A5:** HAI results Season 2016–2017.

ID	Day 0				Day 28				Day 56			
	A/CA/7/09 H1N1	A/Hong Kong/4801/14 H3N2	B/Phuket/3073/13	B/Brisbane/60/2008	A/CA/7/09 H1N1	A/Hong Kong/4801/14 H3N2	B/Phuket/3073/13	B/Brisbane/60/2008	A/CA/7/09 H1N1	A/Hong Kong/4801/14 H3N2	B/Phuket/3073/13	B/Brisbane/60/2008
13	<10	640	<10	<10	640	20480	10	640	640	20480	<10	160
14	40	<10	<10	<10	320	640	<10	<10	640	640	<10	<10
15	<10	<10	<10	<10	<10	<10	<10	<10	80	40	<10	<10
16	320/640	320	<10	<10	640	5120	80	80	1280	2560	80	<10
17	320	40	<10	<10	640	640	80	80	1280	320	40	20
19	<10	320	<10	<10	<10	2560/5120	<10	320	N/S	N/S	N/S	N/S
20	40	80	<10	<10	640	5120	40	80	640	5120	40	40
21	10	<10	<10	<10	160	1280	40	40	320	1280	40	40
22	10	<10	<10	<10	1280	1280/2560	80	320	2560	2560	160	320
23	20	80	<10	<10	320	320	40	320	160	320	40	80/160
24	<10	<10	<10	<10	1280	320	40	320	640	160	10	80/160
25	10	<10	<10	<10	160	160	80	160	320	160	160	160
26	<10	320	<10	<10	320	10240	40	20	320	5120	<10	<10
27	320/640	<10	<10	<10	640	160	<10	20	N/S	N/S	N/S	N/S
28	<10	160	<10	<10	640	320	<10	<10	160	320	<10	<10
29	<10	<10	<10	<10	1280	1280	<10	<10	640	1280	<10	<10
30	<10	2560	<10	<10	<10	5120	80	<10	<10	2560	<10	<10
31	<10	650	<10	<10	<10	640	<10	<10	<10	2560/1280	<10	<10
32	<10	<10	<10	<10	320	640	<10	<10	640	640	<10	<10
33	<10	<10	<10	<10	320	640/1280	20	40	640	640	<10	<10
34	<10	<10	<10	<10	<10	<10	<10	<10	160	<10	<10	<10
35	160	160	<10	<10	640	2560	80	<10	1280	2560	<10	<10
36	<10	320	<10	<10	<10	2560	<10	80	160	2560/1280	<10	<10
38	<10	<10	<10	<10	640	640/1280	80	80	1280	1280	160	<10
39	160	160	<10	<10	640	2560	80	160	640	1280/2560	80	160
40	40	160	<10	<10	320	160	160	40	640	160	80	<10
41	320	<10	<10	<10	2560	<10	<10	<10	2560	160	<10	<10
42	<10	160	<10	<10	2560	1280	320	640	2560	640/1280	320	320
45	640	2560	<10	160	640	160/2560	<10	160	1280	1280/2560	80	160
46	640	1280	<10	<10	1280	2560	<10	320	640	2560	<10	160
47	<10	640	<10	<10	<10	10240	<10	<10	80	10240	<10	<10
48	160	320	<10	<10	1280	2560	160	1280	1280	5120	80	640
49	320	<10	<10	<10	640	640	80	<10	320	640	<10	<10
50	320	1280	<10	<10	1280	2560	80	80/160	640	1280	<10	80
51	320	160	<10	<10	640	2560	<10	80	1280	2560	80	80/160
53	80	<10	<10	80	1280	160	160	<10	N/S	N/S	N/S	N/S
54	320	80	<10	<10	1280	1280	160	320	2560	1280	80	160
55	<10	<10	<10	<10	320	1280	<10	<10	640	2560	<10	<10
57	<10	160	<10	<10	1280	1280/2560	320	80	640	1280	160	40
58	<10	160	<10	<10	320	320	<10	320/640	320	640	80	320
60	320	640	<10	<10	2560	5120	<10	2560/5120	2560	2560	<10	2560
61	<10	1280	<10	<10	160	1280	<10	<10	160	1280	<10	<10
62	<10	320	<10	<10	1280	320	<10	20	640	160	<10	<10
64	<10	<10	<10	<10	160	80	<10	<10	160	160	<10	<10

N/S = no sample.

**Table A6:** HAI results 2017–2018.

ID	Day 0				Day 28				Day 56			
	A/Michigan/45/2015 H1N1	A/Hong Kong/4801/14 H3N2	B/Phuket/3073/13	B/Brisbane/60/2008	A/Michigan/45/2015 H1N1	A/Hong Kong/4801/14 H3N2	B/Phuket/3073/13	B/Brisbane/60/2008	A/Michigan/45/2015 H1N1	A/Hong Kong/4801/14 H3N2	B/Phuket/3073/13	B/Brisbane/60/2008
66	640	160	<10	<10	2560	160	<10	<10	5120	320	<10	<10
67	<10	320	<10	160	2560	1280	160	<10	2560	2560	<10	320/640
68	<10	<10	<10	<10	40	2560	<10	320	2560	1280	<10	640
70	<10	80	<10	<10	1280	640	<10	<10	40	640	<10	<10
71	160	320	<10	<10	160	640	<10	80	1280	640	<10	<10
72	<10	<10	<10	<10	160	10	<10	<10	320	<10	<10	<10
73	<10	<10	<10	<10	10	10	<10	<10	80	40	<10	<10
74	<10	<10	<10	<10	320	80	<10	<10	640	80	80	<10
75	<10	<10	<10	<10	160	80	<10	320	320	160	<10	320
78	<10	320	<10	<10	160	5120	<10	<10	1280	5120	<10	<10
79	320	320	<10	<10	2560	5120	80	<10	2560	2560	80	<10
80	1280	640	<10	160	2560	1280	<10	160/320	640	1280	80	160/320
81	320	<10	<10	<10	640	320	<10	<10	1280	640	80/160	80
83	320	160	10	40	640	10240	160	320	1280	2560	80/160	320
84	1280	1280	80	160	5120	5120	80/160	80/160	2560	1280	160	320
85	320	80	<10	<10	1280	5120	40	80	640	640	<10	<10
86	1280	1280	<10	<10	5120	5120	<10	<10	10240	2560/5120	<10	80
87	160	1280	<10	<10	1280	5120	<10	<10	640/1280	2560	80	<10
88	160	1280	160	80	640	640	320	160	640	1280	160	160
89	5120	<10	<10	<10	2560	320	<10	<10	2560/5120	160	<10	<10
90	80	<10	<10	<10	5120	320	<10	160	5120	320	160	160
92	160	640	<10	<10	5120	2560	<10	160	5120	2560	40/80	80
94	320	<10	<10	<10	640	160	<10	<10	N/S	N/S	N/S	N/S
95	1280	320	<10	<10	640	640	<10	<10	640	320	80	80
96	320/640	320	<10	<10	5120	10240	<10	<10	2560	5120	<10	<10
97	<10	<10	80	<10	160	160	80	<10	160	<10	<10	<10

N/S = no sample.
